# Point Cloud Hand–Object Segmentation Using Multimodal Imaging with Thermal and Color Data for Safe Robotic Object Handover

**DOI:** 10.3390/s21165676

**Published:** 2021-08-23

**Authors:** Yan Zhang, Steffen Müller, Benedict Stephan, Horst-Michael Gross, Gunther Notni

**Affiliations:** 1 Group for Quality Assurance and Industrial Image Processing, Technische Universität Ilmenau, 98693 Ilmenau, Germany; gunther.notni@tu-ilmenau.de; 2 Neuroinformatics and Cognitive Robotics Lab, Technische Universität Ilmenau, 98693 Ilmenau, Germany; steffen.mueller@tu-ilmenau.de (S.M.); benedict.stephan@tu-ilmenau.de (B.S.); horst-michael.gross@tu-ilmenau.de (H.-M.G.); 3 Fraunhofer Institute for Applied Optics and Precision Engineering IOF Jena, 07745 Jena, Germany

**Keywords:** multimodal imaging, thermal, deep neural network, hand segmentation, point cloud segmentation

## Abstract

This paper presents an application of neural networks operating on multimodal 3D data (3D point cloud, RGB, thermal) to effectively and precisely segment human hands and objects held in hand to realize a safe human–robot object handover. We discuss the problems encountered in building a multimodal sensor system, while the focus is on the calibration and alignment of a set of cameras including RGB, thermal, and NIR cameras. We propose the use of a copper–plastic chessboard calibration target with an internal active light source (near-infrared and visible light). By brief heating, the calibration target could be simultaneously and legibly captured by all cameras. Based on the multimodal dataset captured by our sensor system, PointNet, PointNet++, and RandLA-Net are utilized to verify the effectiveness of applying multimodal point cloud data for hand–object segmentation. These networks were trained on various data modes (XYZ, XYZ-T, XYZ-RGB, and XYZ-RGB-T). The experimental results show a significant improvement in the segmentation performance of XYZ-RGB-T (mean Intersection over Union: 82.8% by RandLA-Net) compared with the other three modes (77.3% by XYZ-RGB, 35.7% by XYZ-T, 35.7% by XYZ), in which it is worth mentioning that the Intersection over Union for the single class of hand achieves 92.6%.

## 1. Introduction

Nowadays, robot vision plays an important role in the robotics industry. To enable a robot to navigate or grasp objects as intelligently and safely as a human, a correct understanding of its working environment is a necessary prerequisite. For this task, currently there are many state-of-the-art solutions based on object detection, such as YOLO [1]. However, our work focuses on the vision system of an assistant robot, which is used to transport objects to humans. In order to pick up the object from a human hand without injuring the person, the challenge is achieving exact and efficient pixel-level segmentation and 3D representation of the object and obstacles in the interaction area. In this regard, it is not sufficient to separate hand and object with only a bounding box. Therefore, the discussion in this article will focus on hand–object segmentation.

To solve the segmentation problem, the current mainstream approaches can be classified into two categories.

The first one is color image segmentation based on texture information on the surface of objects. Extensive research has been done on this subject and some of the achievements are impressive, such as the MASK R-CNN network [2] or the PointRend network [3]. However, there are a number of difficulties in hand segmentation, such as the effect of lighting conditions, confusion with objects whose color resembles human skin, and the variety of skin tones.

The second category is 3D point cloud segmentation based on geometric features of objects. In this respect, some challenges such as the articulated nature of the human body, changes in appearance, and partial occlusions [4] make hand segmentation in point clouds more difficult than in RGB images.

Although the deep learning technology has repeatedly surprised in the field of image processing, the abovementioned particular difficulties for hand segmentation can never be solved completely. For example, in [5], the authors explicitly mention that their VGG-16-based [6] hand segmentation network (2D RGB segmentation) can achieve a 91.0% mean IoU (Intersection over Union) on their dataset. If the hand has a complex interaction with other objects, such as holding a complex-shaped object in the hand, it is hard using their approach to detect the hand in the contact areas. Nevertheless, the segmentation of real-world data seen in interactions with humans is just the core challenge for an assistant robot aiming to grasp objects from a human hand.

Since humans are warm-blooded, our body temperature stays almost constant, while skin color, light conditions, and hand posture are varied. Therefore, body temperature is a more stable and robust feature for hand recognition or segmentation compared with RGB data alone. We propose applying an additional LWIR camera (thermal camera) (LWIR, long-wave infrared) to mitigate the problems for hand segmentation mentioned above. However, there are also some difficulties with thermal image segmentation. As mentioned in [7], for an outdoor intelligent surveillance system with a thermal camera, in summer or on a hot day, the contrast of human and background becomes very low and makes it difficult to distinguish human areas from the background in the thermal image. This low contrast problem holds also for a couple of indoor scenarios, e.g., in industrial facilities, where there are differently tempered objects in the background. In addition, an object that is held in hand for a longer time will become similar in temperature to the hand, lowering the contrast to the fingers as well. In this case, hand and object segmentation will also become tough and additional features are needed. In the research of Kim et al. [8], 2D multimodal imaging fusing LWIR and RGB-D images was used for first-person-view hand segmentation, and their results using a DeepLabV3+ [9] network showed that using LWIR obtained 5% better hand IoU performance than using just RGB-D frames.

Therefore, in this work, we will explain a multimodal 3D sensor system composed of a 3D sensor, an RGB camera, and a thermal camera, which is able to capture point cloud data with 7 channels (XYZ-RGB-T). None of these channels are all-purpose, but combined, the information of each channel compensates for their respective weaknesses. It is reasonable to expect that the multimodal 3D data carries more potential features compared with 3D data alone, and that these complex features can be learned by a neural network as well. Besides that, the calibration and registration approach for the sensors will be described. By using this sensor system, a multimodal dataset was captured in order to evaluate the performance of applying the multimodal 3D data for hand and object segmentation. The state-of-the-art methods PointNet, PointNet++, and RandLA-Net were trained and compared on that dataset.

## 2. Related Work

In this section, previous studies on the application of thermal imaging in the field of human recognition will be reviewed. Wang et al. [10] presented a thermal pedestrian detector, in which an edge feature (Shape Context Descriptor) and an Adaboost cascade classifier were adopted. Jeon et al. [7] showed, for an outdoor surveillance thermal camera, that it is hard to segment the human body from the background if the ambient temperature is similar to or higher than the human body temperature (e.g., in summer). To solve this problem, they attempt to perform background subtraction using the sequence of thermal images and a prerecorded background thermal image. However, both of the two studies require a fixed background as a prerequisite, which is not possible for a mobile robot application.

In the research of Setjo et al. [11], Haar cascade classifiers were applied to detect human faces in thermal images and a comprehensive evaluation was conducted with a thermal image dataset comprising a variation of human poses and environmental conditions. They showed that precision and recall of human detection decreases with greater distance to the camera. In addition, the detection results were also affected by the orientation of the face. For such problems, in [12], an integrated analysis for RGB-T (thermal) fusion was proposed to detect human skin using a skin segmentation algorithm (Skindiff) [13]. Their results indicated that the use of the fusion sensor system allows the algorithm to work in environments with many warm objects. An RGB-T dataset and a discussion of the advantages of RGB-T fusion over single RGB or T, such as when objects of interest may not have easily discernible thermal signatures but have strong cues from RGB, can be found in [14]. In addition, there are a number of articles on this topic, such as [15,16,17]. However, all these articles are based on traditional image processing methods. That means that a few parameters or thresholds in the system need to be adjusted manually, and they are usually dependent on the varying camera environment or the state of the camera. For example, in [12], it is mentioned that the response of a thermal camera depends on the uptime of their specific device, which has an effect on the human recognition rate as images become more saturated.

Palermo et al. [4] introduced an RGB-D-T dataset and used HOG (Histogram of oriented gradient) and random forests for human segmentation in an indoor environment. In [18], transfer learning of YOLO [1], a deep learning model, was performed on thermal images for human detection in a night environment. In [19], YOLO was applied on 6-channel 2D images containing RGB color and various geometric features (point density, difference of normal, and curvature). A further CNN (convolutional neural network) model named MCNet for 2D thermal image semantic segmentation of nighttime driving scenes was published in [20]. For point cloud data, in [21] a PointNet-based [22] hand segmentation network is explained, but they do not use multimodal data at all.

In summary, the abovementioned studies can be roughly categorized into three groups:

1 Using traditional methods to analyze multimodal data for human recognition, such as [7,10,14].
2 Using a neural network approach to detect humans but not on multimodal data, such as [5,21].
3 Using a neural network approach to process multimodal data for human detection; however, almost all of them are regarding the task of autonomous driving in urban scenarios, such as [18,20].

To our knowledge, there has not been a comprehensive study using deep learning technology and multimodal 3D data to specifically address the problem of indoor human hand segmentation in point clouds for an assistant robot. Therefore, in this work, we will provide a detailed discussion on this issue.

## 3. Method Overview

**Figure 1:**
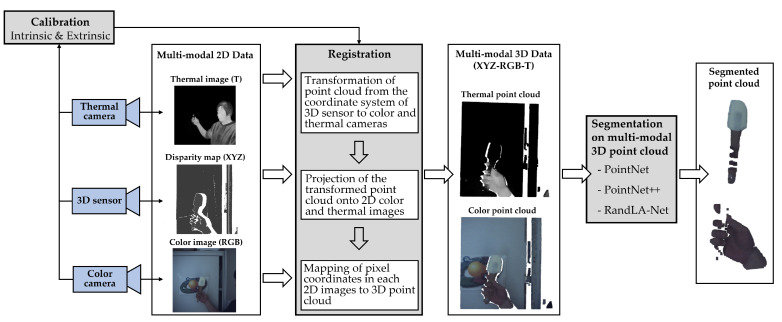
Workflow for a hand–object segmentation approach using a multimodal 3D sensor system containing a 3D sensor, an RGB camera, and a thermal camera.

As shown in Figure 1, the entire pipeline for hand and object segmentation based on multimodal 3D data is divided into 3 steps: Calibration, Registration, and Segmentation.

- **Calibration**: For a multimodal sensor system containing a 3D sensor, a color camera, and a thermal camera, the intrinsic parameters of each sensor and the extrinsic parameters of color and thermal camera with respect to the 3D sensor should be calibrated. This will be explained in Section 4.2.
- **Registration**: By using the intrinsic and extrinsic parameters, the color and thermal pixel values in 2D images should be mapped onto the 3D point cloud in order to build a multimodal 3D point cloud, in which each point integrates multimodal information of color (RGB), temperature (T), and coordinates in 3D space (XYZ). This will be described in Section 4.3.
- **Segmentation**: With the help of neural networks, potential multimodal features hidden in the point cloud can be learned for secure and robust hand–object segmentation. In this work, PointNet [22], PointNet++ [23], and RandLA-Net [24] were used as segmentation approaches. These approaches will be briefly discussed in Section 5. In Section 7, comparative experiments (training on various data modes of XYZ, XYZ-T, XYZ-RGB, and XYZ-RGB-T) will be provided to evaluate the application of multimodal 3D data for hand–object segmentation.

## 4. Sensor System

### 4.1. Multimodal Sensors

**Figure 2:**
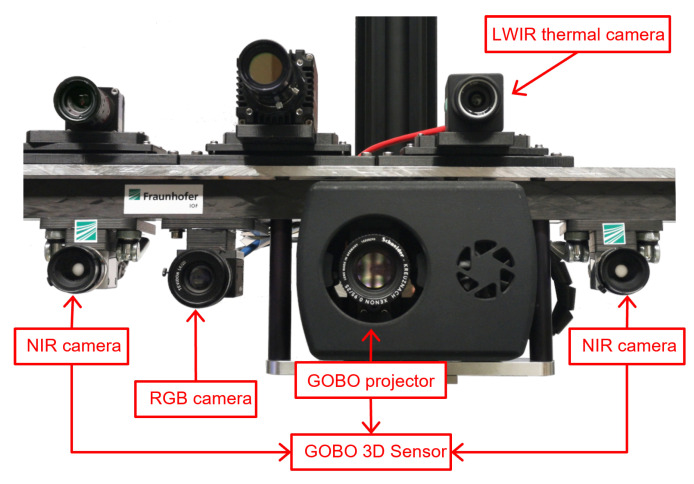
A multimodal 3D sensor system consisting of an active stereovision 3D sensor based on GOBO projection, an RGB camera (FLIR Grasshopper3), and a thermal camera (FLIR A35).

Figure 2 shows our multimodal 3D imaging system, which consists of an active stereovision 3D sensor based on GOBO (Goes Before Optics) projection [25], a color camera (FLIR Grasshopper3 [26]), and a thermal camera (FLIR A35 [27]). It has been used to record a multimodal dataset (XYZ-RGB-T) of humans holding objects, as described in Section 6.

The 3D sensor utilizes two NIR (near-infrared) (850 nm) cameras and a NIR (850 nm) GOBO projector [28] to project a temporally varying aperiodic sinusoidal pattern into the scene. By means of that pattern, corresponding 3D points can be identified in an image sequence, enabling a robust reconstruction of pixel disparities and, therefore, depth of point cloud points. The GOBO system yields point clouds with 0.32–1.18 mm resolution and roughly 0.15 mm measurement error in a relatively small field of view of 48° × 44° in a limited range of 0.4–1.5 m at 36 Hz. The FLIR Grasshopper3 provides color images with a resolution of 2048 × 2048 pixels in the field of view of 50° × 50° at 90 Hz, and the FLIR A35 captures thermal images of 320 × 256 pixels in a range of −25 °C to 135 °C at 60 Hz. It has a field of view of 63° × 50° and therefore covers the whole point cloud area as does the RGB camera. Table 1 shows the technical data of the GOBO 3D sensor and the additional cameras.

### 4.2. Calibration Target

**Figure 3:**
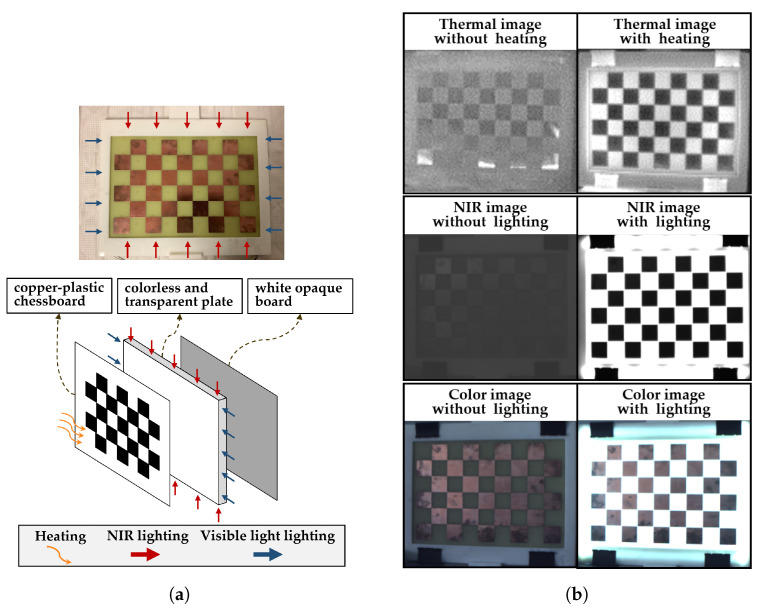
(**a**) A copper–plastic chessboard calibration target (upper) and its principle (bottom). (**b**) Comparison of calibration images with and without active lighting for color image, NIR image, and thermal image.

In order to fuse the image data of each camera, precise mapping of pixel coordinates in each image to the 3D point cloud is required and the camera system needs to be calibrated. Normally, a printed checkerboard pattern can be used as a calibration target. It works fine for RGB and NIR cameras, but for the thermal camera, it is challenging. As black and white grids of the printed pattern have almost the same emittance in LWIR, the chessboard pattern cannot be captured by thermal cameras. Hence, as shown in Figure 3a, inspired by [30], a copper–plastic chessboard calibration target has been manufactured to solve this problem. Before the actual calibration, the target needs to be heated, for example, by means of a hair dryer. After a few seconds of cooling down, the copper grids and plastic grids will have different temperatures and different gray values (copper dark and plastic bright) in the thermal image, because they have different emissivity coefficients, as shown in Figure 3b.

This copper–plastic chessboard works fine for thermal camera calibration, but it brings another problem for RGB and NIR camera. The surface of copper plating is always smooth, resulting in an overexposure problem because of specular reflections with external and passive light sources. Furthermore, the low contrast of texture on the chessboard surface in the wavelength of visible light and NIR leads to the fact that the grid in the calibration images is not sharp enough for the corners to be detected. Therefore, as shown in Figure 3a, a calibration target with an internal active light source is proposed. A colorless and transparent plate is mounted behind the chessboard and visible light, and NIR LEDs are mounted at the edges of the plate as active light sources. A white, opaque board is set behind the plate as a diffuser. Figure 3b shows the comparison of calibration images with passive lighting and active lighting. With the help of the active light source, images with sufficient contrast can be captured to calibrate the intrinsic and extrinsic parameters for our multimodal cameras.

### 4.3. Calibration and Registration

The intrinsic parameters K of each camera can be simply calculated using Zhang’s calibration algorithm [31]. The 3D sensor is used as a reference camera for calibration of extrinsic parameters. This means that rotation R and translation T of each camera (except 3D sensor) with respect to the coordinate system of the 3D sensor are calculated from a series of image tuples showing the calibration target. By using the parameters, alignment of the multimodal point cloud can be performed with the following method:

For generating the multimodal point cloud, each point of the original point cloud from the 3D sensor is projected onto the image plane of the color and thermal camera. To that end, the point cloud can be transformed from the coordinate system of the 3D sensor to the coordinate system of the target camera with the extrinsic parameters $\left(R_{t},T_{t}\right)$. For each 3D point of the transformed point cloud, a 2D projection pixel $\left(u_{t},v_{t}\right)$ on the target sensor plane can be calculated with intrinsic parameter $K_{t}$ of the thermal camera and RGB camera, respectively. If the projection pixel is located on the sensor, i.e., $0\leq u_{t}<width$, $0\leq v_{t}<height$, it will be determined as a corresponding pixel of this 3D point, as shown in Equation (1). Once the corresponding 2D pixels of all 3D points are determined, thermal and RGB values can be mapped onto the 3D point cloud.

(1) $$s\cdot \left[\begin{array}{c} u_{t}\\ v_{t}\\ 1 \end{array}\right]=K_{t}\cdot \left(\left[\begin{array}{ccc} r_{11} & r_{12} & r_{13}\\ r_{21} & r_{22} & r_{23}\\ r_{31} & r_{32} & r_{33} \end{array}\right]\cdot \left[\begin{array}{c} x\\ y\\ z \end{array}\right]+\left[\begin{array}{c} t_{x}\\ t_{y}\\ t_{z} \end{array}\right]\right),\;K_{t}=\left[\begin{array}{ccc} f_{x} & 0 & c_{x}\\ 0 & f_{y} & c_{y}\\ 0 & 0 & 1 \end{array}\right]$$

where $K_{t}$ is the matrix of intrinsic parameters of target camera, $c_{x}$ and $c_{y}$ are the principal point coordinates, $f_{x}$ and $f_{y}$ are the focal lengths of thermal or RGB camera’s lens, $r_{ij}$ represent the rotation matrix, and $\left[t_{x},t_{y},t_{z}\right]$ is the translation vector defining the extrinsic calibration parameters. *x*, *y*, and *z* are the point coordinates in the coordinate system of the 3D sensor.

## 5. Point Cloud Segmentation Networks

### 5.1. PointNet

**Figure 4:**
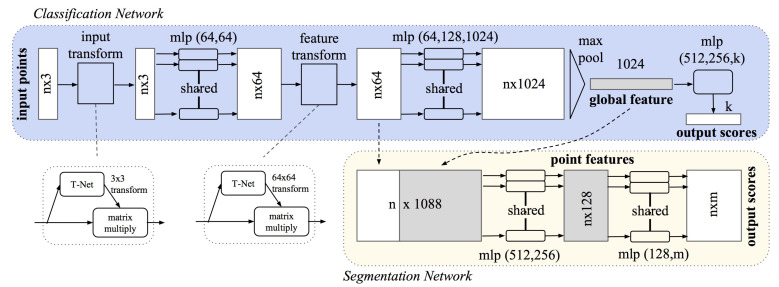
The principle of PointNet. Point positions are transformed into spatial features by two stages of MLPs, before they are pooled into a global feature vector describing the whole object. Afterwards, a combination of local and global features can be used for segmentation purposes [22].

In the field of 3D point cloud segmentation, PointNet [22] is a milestone study. The article proposed the idea of using shared multilayer perceptrons (MLP) to extract global features from a point cloud. By using a novel T-Net (transformation-network), a reference frame for the point cloud can be learned and utilized to keep features rotationally invariant. In traditional methods, principal component analysis (PCA) was usually used to solve this problem instead. In addition, max-pooling was recommended as a symmetric aggregation function to solve the problem that usually, a point cloud is an unordered set. As shown in Figure 4, global features of a point cloud can be efficiently extracted using PointNet. Finally, the global features and the output features of the last feature transformation unit will be concatenated to be used as input for another network to achieve pixel-level segmentation.

### 5.2. PointNet++

**Figure 5:**
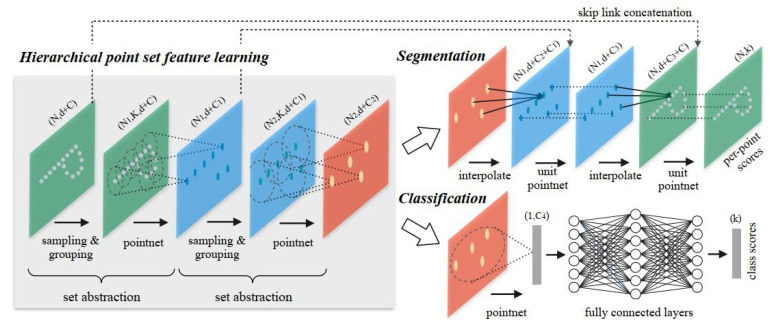
The multilevel architecture of PointNet++. Explicit neighborhood search in the point clouds is used to extract local features by means of a locally applied PointNet in multiple stages. These strong local feature can be used for object classification (lower branch) or for segmentation (upper branch) [23].

Obviously, with only global features, PointNet has insufficient ability to represent semantic information for a local region. PointNet++ [23] describes a multilevel architecture, as shown in Figure 5. By using a farthest point sampling (FPS) algorithm, in each level, the input point cloud is progressively downsampled and the point density decreases. Each point in the sampled sparse point cloud is used as a centroid for a neighborhood search in the dense point cloud. Then, a mini-PointNet is utilized to extract the global features of this neighborhood that will be used as the local feature of this centroid point. A hierarchical propagation strategy with distance-based interpolation and across level skip links is adopted to upsample the enriched point clouds to the original size.

### 5.3. RandLA-Net

**Figure 6:**
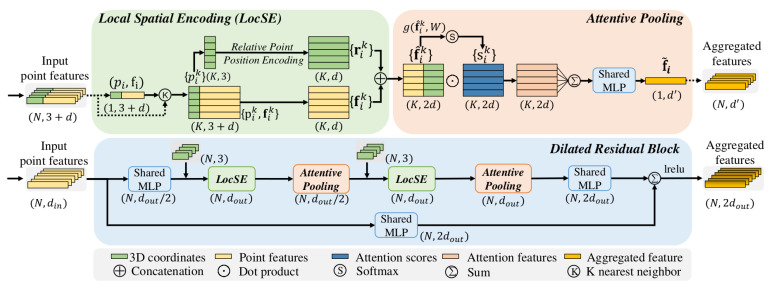
The architecture of the local feature aggregation module of RandLA-Net, which consists of multiple Local Spatial Encoding layers (LocSE) and Attentive Pooling layers (AP). In LocSE, the geometric information of a local area in the point cloud is encoded and then concatenated with the point features for local feature extraction. The local features are further aggregated by an AP layer [24].

RandLA-Net [24] is a state-of-the-art neural network designed for large-scale 3D point cloud semantic segmentation. Similar to PointNet++, RandLA-Net is also a multilevel architecture, which, in contrast, uses random downsampling instead of FPS in order to reduce memory requirements and speed up computation. However, random sampling has the drawback of missing some useful point features occasionally. To overcome that issue, a powerful local feature aggregation module was designed in that approach, as shown in Figure 6. By using a local spatial encoding module (LocSE) in each neighborhood, various spatial information are explicitly concatenated and encoded. Therefore, XYZ-coordinates of all points as well as euclidean distances and XYZ-differences between the centroid point and all neighboring points are calculated. Then, the spatial information and point features are concatenated and local features can be extracted using a shared MLP. Additionally, between two adjacent levels, attentive pooling is utilized to aggregate the features. Then, multiple LocSE and attentive pooling units with a skip connection are stacked as a dilated residual block, which is repeatedly used in the RandLA-Net. Overall, RandLA-Net is built by stacking multiple dilated residual blocks to aggregate local features, and an upsampling method identical to PointNet++ is used to interpolate the downsampled point clouds.

## 6. Datasets for Hand–Object Segmentation

**Figure 7:**
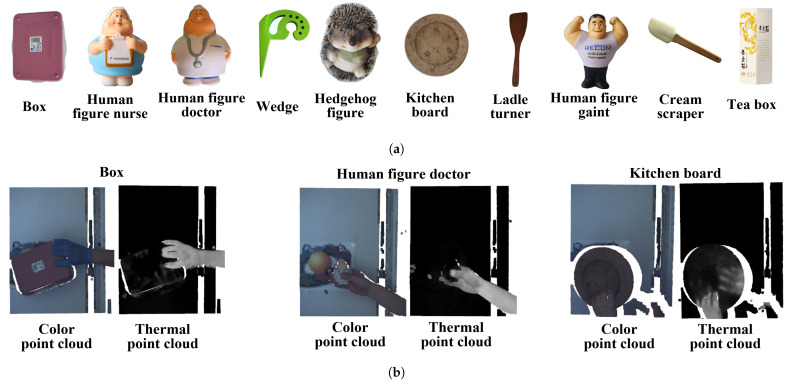
Overview of the GOBO-Dataset with 12 classes (10 objects, background, and hand): (**a**) all objects; (**b**) examples of multimodal 3D data (Box, Human figure doctor, and Kitchen board).

In this paper, a dataset captured by our sensor system and named GOBO-Dataset is used to evaluate the performance of the multimodal 3D data hand–object segmentation, as shown in Figure 7. The hand with one of the objects was placed roughly one meter in front of the sensors. In half of the data, the human hand is recorded with opaque rubber gloves; in the other half, without. In some samples of the dataset, the objects have taken the temperature of the holding hand caused by the long time holding them (see the right thermal point cloud). We used our own semiautonomous annotation tool for labeling these multimodal point clouds. The tool takes advantage of the simple separation of the background in the point cloud and uses region growing on the thermal or color channel for an initial separation of the hand and held object, which afterwards can be refined manually.

The GOBO-Dataset provides 600 multimodal point clouds labeled with 12 classes (10 objects, background, and human hand). The samples have been split into a training set with 420 point clouds, a validation set with 60 point clouds, and a test set with another 120 point clouds. In the dataset, we have multimodal point clouds with 7-channels containing spatial data (XYZ), color data (RGB), and thermal data (T). In comparative experiments, the networks mentioned in Section 5 were trained on different modalities of the dataset in order to understand the influence of the individual parts (XYZ, XYZ-RGB, XYZ-T, and XYZ-RGB-T).

## 7. Segmentation Experiment

As mentioned in Section 5, PointNet has the simplest architecture with only plain global feature extraction. Local features can be extracted by PointNet++ and RandLA-Net; especially, RandLA-Net has a more powerful and complex local feature aggregation with respect to PointNet++. Therefore, in this experiment we have chosen these three networks with different performances for training on the GOBO-Dataset to evaluate the influence of multimodal 3D data on hand–object segmentation in general. Thus, the findings should generalize to future architectures.

### 7.1. Evaluation Approach

A measuring method is required for performance evaluation of the point cloud segmentation. The Intersection over Union (IoU) was applied to intuitively reflect segmentation performance. For each class, the IoU can be calculated using Equation (2). Moreover, the mean IoU of all the classes (hand and objects) was calculated to present the overall performance, and the mean IoU of all the objects has been also provided in the experiment section. In the scene of grabbing an object from a human hand by an assistant robot, we did not measure the IoU for background because the segmentation of hand and held object is much more important than background, while the background class otherwise dominates the results.

(2) $$\mathrm{IoU}=\frac{TP}{TP+FP+FN}$$

where TP is the number of true-positive predicted point classes, FP is the number of false-positive predictions, and FN is the number of false-negative predictions.

### 7.2. Training Details

PointNet, PointNet++, and RandLA-Net were trained on XYZ, XYZ-RGB, XYZ-T, and XYZ-RGB-T for 400 epochs without any pretraining. The learning rate setting is as shown in Table 2. We used a framework of PointNet and PointNet++ available from [32]. For PointNet++, for the sake of efficiency, we replaced the farthest point sampling by a uniform random sampling similar to RandLA-Net, and multiscale grouping (MSG) was also adopted. The used implementation of RandLA-Net is can be found in [33]. For PointNet++ and RandLA-Net, a 4-level architecture was used, and in each level, the point cloud was progressively downsampled with a factor of 1/3.

Due to removal of invalid points, 3D sensors will inevitably produce point clouds of different sizes and, depending on the scene, the number of valid points varies. However, neural networks require batches of data with the same size for training. Hence, before training, the multimodal point clouds in the training dataset were uniformly downsampled to standardized size (10,000 points in our case).

The training of the networks used the cross-entropy loss function and the Adam optimizer [34]. The point clouds in our dataset are imbalanced in the number of points per class (the ratio of background, hand, and object is approximately 3309:267:1). Therefore, different weights for each class were used for weighting the loss function, as shown in Equation (3). The normalized weight $w_{i}$ depends on the probability $p_{i}$ of a point to belong to the the *i*th of *K* classes in the entire dataset.

(3) $$w_{i}=\frac{log(p_{i})}{\sum_{j=0}^{K}log\left(p_{j}\right)}$$

### 7.3. Segmentation Results

**Figure 8:**
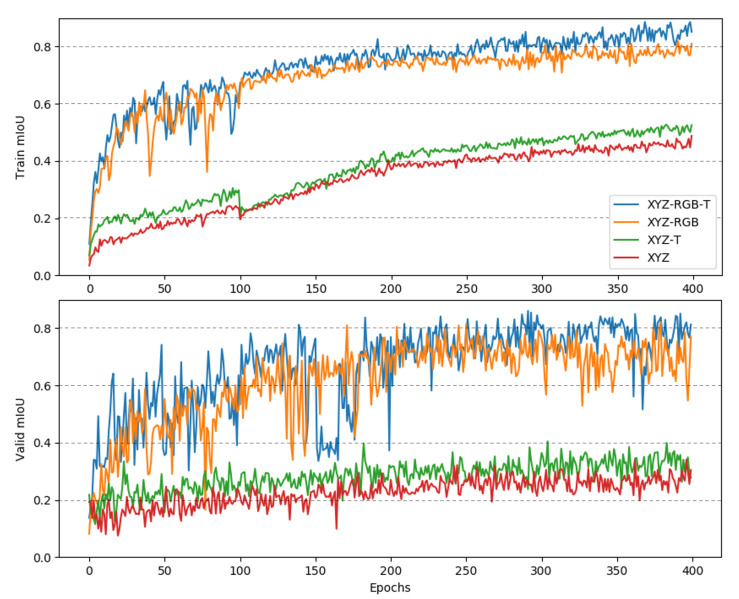
The convergence curves in training phase of RandLA-Net, in which RandLA-Net was trained for 400 epochs on data XYZ, XYZ-T, XYZ-RGB, and XYZ-RGB-T. Upper—the training curve; bottom—the validation curve.

Figure 8 shows convergence curves of RandLA-Net in the training phase on the training dataset and validation dataset. At epoch 400, although the training curve still shows an improving trend, the validation curve indicates that the results are no longer improving. So, we interrupted the training at the 400th epoch. Obviously, with the help of the strong feature of color, the RandLA-Net has a significant superiority by XYZ-RGB-T and XYZ-RGB over XYZ and XYZ-T. With the use of thermal, XYZ-RGB-T has further improved over XYZ-RGB. This is in line with our expectation. Meanwhile, trends of the convergence curves show that the convergence rate of the four modes were almost the same. This indicates that the multimodal point cloud does not lead to a longer training time due to more channels. The training phases of PointNet and PointNet++ feature almost the same tendency.

**Figure 9:**
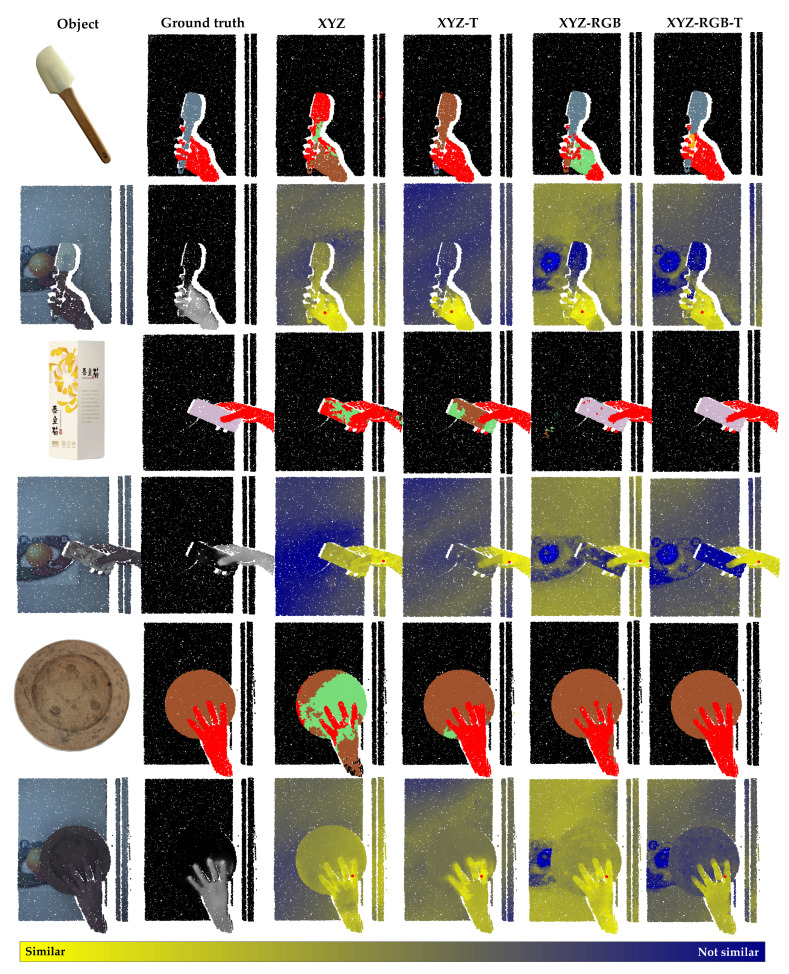
Visualization of experimental results for RandLA-Net on individual samples of the test dataset: The first row shows the ground truth and segmentation by XYZ, XYZ-T, XYZ-RGB, and XYZ-RGB-T, while the hand class is labeled in red. The second row shows the color point cloud, thermal point cloud, and the feature point cloud generated by XYZ, XYZ-T, XYZ-RGB, and XYZ-RGB-T. In the feature point cloud, the euclidean distances between a reference point (red point) and all other points are calculated and normalized in features space. The distances are color coded (light yellow—similar points, dark blue—dissimilar points).

Table 3 shows the detailed quantitative segmentation results based on the test dataset. For all three networks, the overall mIoU shows similar relations for the individual input channels used, while the absolute performance of the three networks differs, reflecting their individual abilities. However, XYZ-T has almost no improvement over XYZ in the test dataset independent of the used architecture. For this, the second and third columns of the table provide the explanation. For example, for RandLA-Net, the mIoU of objects by XYZ-T actually decreased by 0.7% compared with XYZ. It is possible that this is because in some samples of our dataset, the object took on hand temperature at some parts of the surface. The points in these areas could be confused for hand without any additional color information. Compared to XYZ-RGB, although XYZ-RGB-T should not have a dominant advantage for predicting the object points, it has a significantly better object mIoU. This indirect improvement is due to the reduction of false-positive points in the interaction area of hand and object, which can be better predicted as hand, as shown in Figure 9. In comparison, the object mIoU, as well as the mIoU of the hand class, has an obvious improvement from XYZ to XYZ-RGB-T, proving that multimodal data significantly supports a more robust segmentation independent of the actual method used.

### 7.4. Visualization of Segmentation Results

Figure 9 shows a visualization of the segmentation results. For each object, the first row shows the ground truth and RandLA-Net predictions by XYZ, XYZ-T, XYZ-RGB, and XYZ-RGB-T. The second row shows the color point cloud, thermal point cloud, and feature point clouds. The features extracted by the last feature layer of RandLA-Net were used to generate these feature point clouds. Inspired by [35], we used the following method to generate the feature point cloud:

First, we chose a reference point (red point) located on the hand. In the corresponding feature space, the euclidean distances between this reference point and all the other points of this point cloud were calculated. The 3D sensor inevitably will generate some outlier points (incorrectly reconstructed points). In feature space, the distances between these points and other points may be exceptionally large. Therefore, the distances for visualization were normalized to the 97% quantile and presented with gradient colors (light yellow to dark blue). Hence, in the feature point cloud, the greater color contrast between two points indicates that they have greater dissimilarity.

It is clearly visible that, for all the objects, the feature point cloud of XYZ-RGB-T has higher contrast than any other, i.e., the points of the hand have greater distances in the feature space to the object and background. Although the final segmentation result is still dependent on the classifier, these distances make it easier to cluster points in the feature space and implies that the segmentation will be better. Figure 9 shows that XYZ-RGB-T has the best segmentation results in the interaction area of hand and object. For example, in the pictures of the first object, the segmentation of the fingers and the object is refined when using XYZ-RGB-T. For the second object, some areas of the surface possess a similarity to the hand in the feature point cloud by XYZ-RGB because of the color texture. The segmentation results by XYZ-RGB show that some points on these areas are indeed predicted as hand. In comparison, the segmentation of XYZ-RGB-T is much more precise. For the third object, as shown in the thermal point cloud, the boundary area of the kitchen board has a similar temperature as the hand, causing the points in this area to be predicted as hand when using XYZ-T. In contrast, this similar temperature does not affect the prediction when using XYZ-RGB-T. However, the pictures of the third object show that the middle finger of the hand with a ring has points that were mistakenly predicted as object by XYZ-RGB and XYZ-RGB-T classifiers.

### 7.5. Time Consumption Analysis

The experiment was conducted on the computing platform of an Intel Core i9-9960x (CPU) and GeForce RTX 2080 Ti (GPU). We recorded the time consumption for processing a multimodal point cloud with 10 k points. By using a parallel computing by OpenMP [36], multimodal data fusion consumes 14 milliseconds (ms) approximately, and the inference time consumption by the three networks PointNet, PoineNet++, and RandLA-Net are approximately 7 ms, 124 ms, and 102 ms, respectively. As we can see, with respect to the inference by PointNet++ and RandLA-Net, data fusion occupies only a fraction of the time consumption for the entire process. PointNet++ and RandLA-Net have the multilevel architecture, meaning that multiple k-nearest-neighbors (KNN)-based neighborhood searches are required for each of two adjacent levels. As a result, these two approaches are not as efficient as PointNet. The neighborhood search for the 4-level architecture has a time consumption of 81 ms and, hence, is the major part. The additional effort to achieve an improvement through sensor fusion seems to be justified in view of the run-times of increasingly complex networks necessary to improve the results otherwise.

## 8. Discussion

To enable precise segmentation of hand and object for an assistant robot to grasp objects from a human hand safely, in this work, we presented a multimodal 3D sensor system. We also focused on the challenges for calibration and alignment of a multimodal sensor system with a thermal camera. The successful experiments showed that applying a copper–plastic chessboard calibration target with an internal and active light source (NIR and visible light) effectively solves the calibration problem. As it can be captured by each camera with sufficient contrast simultaneously, the use of such a calibration target makes the calibration and alignment of multimodal camera systems no longer tedious.

The segmentation experiments using PointNet, PointNet++, and RandLA-Net on our dataset could confirm our hypothesis that multimodal data significantly supports pointwise segmentation. RandLA-Net, as the strongest state-of-the-art network, has achieved remarkable results on XYZ-RGB-T (overall mIoU: 82.8%). In contrast, the mIoUs for XYZ, XYZ-T, and XYZ-RGB were 35.7%, 35.7%, and 77.3%, respectively. Surprisingly, XYZ-T has almost no improvement over XYZ; this is partly because some objects have a similar temperature to human hands, which confuses the prediction on XYZ-T without any additional cues. In addition, a visualization of feature point cloud extracted by RandLA-Net intuitively demonstrates the feasibility of using a neural network to extract the potential features of multimodal data. As mentioned in Section 1, in the multimodal data, none of the channels are all-purpose, but the information of all channels can be integrated to make up for their respective weaknesses.

In recent years, the computing performance of computers has improved tremendously. Therefore, deep learning technology has started to be widely studied and applied. Under this condition, the computing performance required for efficient multimodal sensor fusion is also achievable. On our computing platform of an Intel Core i9-9960x (CPU) and GeForce RTX 2080 Ti (GPU), data fusion consumes approximately 14 ms for a point cloud with 10 k points. Therefore, we propose the application of multimodal data to reduce the complexity of image processing tasks. It is a matter of data and improvements in the segmentation methods, which, in future, will allow for raising the limits of hand–object segmentation results further. Nevertheless, for safety-critical applications, the IoU results alone will not be a sufficient criterion. In order to rely on machine-learning-based safety-critical features, other questions like explainability and robustness in case of adversarial or out-of-distribution data have to be considered. We are sure that multimodal data help to reach an acceptable level of robustness more easily either way.

In the future, in order to make the multimodal sensor system usable in a real-world environment, we would like to expand our dataset and further evaluate it in practical scenarios. Currently, with training of RandLA-Net on our dataset, we can precisely segment hand and objects in real-time point clouds from the sensor. Nevertheless, some points on the background will be identified as hand or object occasionally, which may be caused by the different point density compared with the training data. Therefore, we address the portability of our models to other sensor setups and unseen objects in future work.

## Figures and Tables

**Table 1:** Technical data of the used camera systems.

	GOBO 3D Sensor	FLIR A35 Thermal Camera	FLIR Grasshopper3 Color Camera
Resolution	1024 × 1024	320 × 256	2048 × 2048
Image frequency	36 Hz	60 Hz	90 Hz
Field of view	48° × 44°	63° × 50°	50° × 50°
Mean depth error	0.15–0.5 mm [29]	-	-
Range	0.4–2 m	-	-
Wavelength band	850 nm	8–14 μm	R—640, G—525, B—470 (nm)
Thermal sensitivity	-	<0.05 °C	-
Temperature range	-	−25 to 135 °C	-

**Table 2:** Learning rate schedule for the experiments.

Epochs	0–100	100–200	200–400
learning rate	0.01	0.001	0.0001

**Table 3:** The quantitative segmentation results on test split of the GOBO-Dataset (IoU %).

		mIoU	IoU	mIoU	Box	Nurse	Doctor	Wedge	Hedge-	Kitchen	Spatula	Human	Ice	Tea
		Overall	Hand	Object		Figure	Figure		hog	Board		Figure	Scraper	Box
RandLA-Net	XYZ-RGB-T	**82.8**	**92.6**	**81.9**	91.7	77.5	65.9	80.3	71.6	85.2	70.7	95.3	88.8	91.5
	XYZ-RGB	77.3	88.9	76.2	77.0	59.9	33.0	80.0	96.2	83.8	68.8	88.1	91.5	83.4
	XYZ-T	35.7	84.1	30.9	44.2	15.0	5.0	28.4	57.7	47.9	27.0	25.3	26.5	32.1
	XYZ	35.7	76.7	31.6	34.3	2.5	36.1	29.4	49.6	65.4	17.8	28.2	22.5	30.6
PointNet++	XYZ-RGB-T	**55.0**	**79.8**	**52.5**	58.0	40.2	33.7	65.9	87.1	71.7	25.5	43.1	24.1	75.2
	XYZ-RGB	45.5	66.6	43.4	66.2	30.2	23.0	58.0	69.7	57.0	23.6	41.1	19.3	46.1
	XYZ-T	25.9	52.0	23.2	45.2	13.3	1.8	20.5	37.6	38.3	15.1	5.5	12.0	43.1
	XYZ	25.8	60.6	22.4	55.1	15.6	12.7	7.8	27.8	43.3	24.3	6.4	15.8	14.7
PointNet	XYZ-RGB-T	**45.9**	**79.9**	**42.5**	62.2	23.5	26.7	62.6	36.2	52.7	28.8	39.3	42.2	50.9
	XYZ-RGB	43.5	78.4	40.1	60.1	20.5	22.6	63.5	34.9	51.8	27.0	38.6	42.9	39.2
	XYZ-T	24.8	72.1	20.1	18.1	31.4	13.9	16.1	25.3	23.6	9.1	26.3	18.9	18.2
	XYZ	22.0	52.9	18.9	36.4	15.8	13.9	10.4	24.0	31.0	12.9	18.1	11.6	15.2
